# Intensity Thresholds on Raw Acceleration Data: Euclidean Norm Minus One (ENMO) and Mean Amplitude Deviation (MAD) Approaches

**DOI:** 10.1371/journal.pone.0164045

**Published:** 2016-10-05

**Authors:** Kishan Bakrania, Thomas Yates, Alex V. Rowlands, Dale W. Esliger, Sarah Bunnewell, James Sanders, Melanie Davies, Kamlesh Khunti, Charlotte L. Edwardson

**Affiliations:** 1 Department of Health Sciences, University of Leicester, Leicester General Hospital, Leicester, Leicestershire, United Kingdom; 2 Diabetes Research Centre, University of Leicester, Leicester General Hospital, Leicester, Leicestershire, United Kingdom; 3 National Institute for Health Research (NIHR) Leicester-Loughborough Diet, Lifestyle and Physical Activity Biomedical Research Unit (BRU), Diabetes Research Centre, Leicester General Hospital, Leicester, Leicestershire, United Kingdom; 4 Alliance for Research in Exercise, Nutrition and Activity (ARENA), Sansom Institute for Health Research, Division of Health Sciences, University of South Australia, Adelaide, Australia; 5 School of Sport, Exercise and Health Sciences, Loughborough University, Loughborough, Leicestershire, United Kingdom; 6 National Institute for Health Research (NIHR) Collaboration for Leadership in Applied Health Research and Care – East Midlands (CLAHRC – EM), Diabetes Research Centre, Leicester General Hospital, Leicester, Leicestershire, United Kingdom; Vanderbilt University, UNITED STATES

## Abstract

**Objectives:**

(1) To develop and internally-validate Euclidean Norm Minus One (ENMO) and Mean Amplitude Deviation (MAD) thresholds for separating sedentary behaviours from common light-intensity physical activities using raw acceleration data collected from both hip- and wrist-worn tri-axial accelerometers; and (2) to compare and evaluate the performances between the ENMO and MAD metrics.

**Methods:**

Thirty-three adults [mean age (standard deviation (SD)) = 27.4 (5.9) years; mean BMI (SD) = 23.9 (3.7) kg/m^2^; 20 females (60.6%)] wore four accelerometers; an ActiGraph GT3X+ and a GENEActiv on the right hip; and an ActiGraph GT3X+ and a GENEActiv on the non-dominant wrist. Under laboratory-conditions, participants performed 16 different activities (11 sedentary behaviours and 5 light-intensity physical activities) for 5 minutes each. ENMO and MAD were computed from the raw acceleration data, and logistic regression and receiver-operating-characteristic (ROC) analyses were implemented to derive thresholds for activity discrimination. Areas under ROC curves (AUROC) were calculated to summarise performances and thresholds were assessed via executing leave-one-out-cross-validations.

**Results:**

For both hip and wrist monitor placements, in comparison to the ActiGraph GT3X+ monitors, the ENMO and MAD values derived from the GENEActiv devices were observed to be slightly higher, particularly for the lower-intensity activities. Monitor-specific hip and wrist ENMO and MAD thresholds showed excellent ability for separating sedentary behaviours from motion-based light-intensity physical activities (in general, AUROCs >0.95), with validation indicating robustness. However, poor classification was experienced when attempting to isolate standing still from sedentary behaviours (in general, AUROCs <0.65). The ENMO and MAD metrics tended to perform similarly across activities and accelerometer brands.

**Conclusions:**

Researchers can utilise these robust monitor-specific hip and wrist ENMO and MAD thresholds, in order to accurately separate sedentary behaviours from common motion-based light-intensity physical activities. However, caution should be taken if isolating sedentary behaviours from standing is of particular interest.

## Introduction

There is cumulative evidence that sedentary behaviour, characterised as any waking behaviour with low energy expenditure (≤1.5 metabolic equivalents) while in a sitting or reclining posture [1], is detrimentally associated with a number of health outcomes including cardiovascular disease, type 2 diabetes mellitus and all-cause mortality [2–7]. This has important implications given that adults spend the majority of their waking hours (~55% to ~70%) sedentary [8–11]. Correspondingly, engaging in light-intensity physical activities (e.g. standing and light walking) has been shown to have beneficial effects on health [12–14]. Therefore, accurately identifying and distinguishing between sedentary behaviour and light-intensity physical activity is extremely important. Tri-axial accelerometers, which quantify the acceleration and deceleration in orthogonal directions of three dimensional space, have gained a reputation as the preferred method of collecting objective measurements of physical activity and sedentary behaviour data in health research [15, 16]. These devices have the ability to accumulate large amounts of acceleration data (usually over an adjustable sampling frequency range) that can be translated into physical activity and sedentary behaviour parameters (i.e. duration, frequency and intensity) [17].

Accelerometers have historically provided data in the form of ‘counts’—an aggregate measure of the intensity and magnitude of accelerations over a given time epoch [18, 19]. Count-based systems are straightforward to operate and do not expend substantial amounts of computational memory. However, counts are produced via proprietary algorithms which are developed and patented by the manufacturers of these monitors (entailing different amplifiers, filters, frequencies, etc.) [18, 19]. Therefore, even if the same reference acceleration signal is being measured, different devices can produce diverse count values [19]. This makes it difficult to equate data between different accelerometer brands and thus, problematic to compare results from studies that have employed different devices. However, due to the significant improvements in technologies over the last few years, raw acceleration data can now be measured and stored at high frequencies, with no need to summarise into proprietary count-based epochs [19–24]. As a consequence, there is a necessity for the analysis of raw acceleration data using approaches that can be understood and used by all.

The challenge of analysing raw signals revolves around several factors: the management of vast amount of data which are generated; the requirement to remove the gravitational and noise components incorporated within the signals [25]; and the requirement of feasible mathematical and/or statistical tools to accurately analyse and make valid interpretations from the data. Procedures for processing the raw acceleration data and attempting to separate the movement and gravitational components of the signal include the: Signal Magnitude Area (SMA) [26–28], Euclidean Norm Minus One (ENMO) [25, 29, 30] and Mean Amplitude Deviation (MAD) methods [31–33]. The SMA can be calculated after applying computationally expensive mathematical filters (e.g. Butterworth high pass filters, etc.) in order to remove the gravitational component [26–28]. In contrast, the recently proposed ENMO and MAD metrics do not require the data to be filtered in order to correct for gravity—since they systematically take this element into account within their algorithms [25, 29–33], making these particular analytical techniques attractive. For example, MAD represents the mean value of the dynamic acceleration component. It is computed from the resultant vector value of the measured orthogonal acceleration, which involves a dynamic component due to deviations in velocity, and a static element due to gravity. The static element is removed from the analysed epoch and the remaining dynamic component is revised. Thus, the MAD value can be regarded as the mean of the revised acceleration signal autonomous of the static element within the epoch. The ENMO metric, which is also computed from the resultant vector of the measured orthogonal acceleration, adjusts for gravity via subtracting a fixed offset of one gravitational unit from the Euclidean Norm of the three raw acceleration signals. Therefore, ENMO, which can also be regarded as the revised acceleration signal autonomous of the static gravitational element, equally signifies the dynamic acceleration component.

To our knowledge, only three studies have methodically investigated the use of the MAD metric with raw acceleration data relating to physical activity [31–33]. Vähä-Ypyä and colleagues recently derived MAD-based universal thresholds for differentiating sedentary and standing activities from walking and different speeds of bipedal movement [33]. Although these are useful, they are not beneficial for researchers focusing on the time spent in sedentary behaviours (e.g. lying/sitting) and common light-intensity physical activities (e.g. washing pots, dusting, etc.). Besides the ActiGraph GT3X device [30 Hz; ActiGraph Corporation, Pensacola, Florida, United States of America], the thresholds were defined for unconventional devices (Hookie AM13 [100 Hz; Hookie Technologies Ltd, Espoo, Finland] and Gulfcoast X6-1A [20 Hz; Gulf Coast Data Concepts LLC, Waveland, Mississippi, United States of America]) that are not in widespread use. Vähä-Ypyä and colleagues proceeded to develop universal thresholds applicable to raw acceleration data collected from these monitors worn at the hip; however, as significantly large differences in MAD values were evident between accelerometer brands, these should be used with caution.

Accelerometers were traditionally worn on the hip, however, in recent years wrist-worn accelerometry has emerged and is now also being used in large national health surveys (e.g. NHANES [34] and UK Biobank [35]). Therefore, it is essential to also develop analytical methods which are appropriate for use with data from wrist-worn monitors and can be applied to existing methods for processing raw acceleration data (e.g. ENMO and MAD). Furthermore, to date, the MAD metric has not been compared to ENMO [31–33], which is emerging as the model metric for efficiently analysing raw acceleration data and classifying intensity [25, 29, 30]. Although ENMO thresholds to classify moderate and vigorous physical activities using raw acceleration data have previously been developed [29], ENMO thresholds to separate sedentary behaviours from light-intensity physical activities have yet to be proposed.

Therefore, by using raw acceleration data collected from both hip- and wrist-worn widely-used tri-axial accelerometers, our aims are to: (1) extend the premise of the ENMO and MAD metrics via developing internally-validated monitor-specific intensity-based thresholds for discriminating between sedentary behaviours and common light-intensity physical activities; and (2) compare and evaluate the performances between the ENMO and MAD metrics. The generation of ENMO and MAD thresholds developed will be sample and protocol specific. Hence, the classifications of sedentary behaviours and light-intensity physical activities should be broadly comparable between studies—irrespective of the metric, accelerometer brand and wear-site used.

## Methods

### Study Sample

Investigations were carried out using data from a laboratory-based study which was conducted by the National Institute for Health Research (NIHR) Leicester-Loughborough Diet, Lifestyle and Physical Activity Biomedical Research Unit (BRU). The study was implemented within a bespoke laboratory, located at Loughborough University (Loughborough, Leicestershire, United Kingdom), which was furnished to enable the sedentary and non-sedentary tasks to be undertaken efficiently. Participants (aged ≥ 18 years) were recruited via email and word of mouth. All participants provided written informed consent, and the study was approved by the Ethics Committee of Loughborough University.

### Accelerometer Devices

Two distinct and commercially available tri-axial accelerometers were utilised: the ActiGraph GT3X+ monitor (dynamic range: ± 6*g*, sampling frequency range: 30–100 Hz [ActiGraph Corporation, Pensacola, Florida, United States of America]) and the GENEActiv Original monitor (dynamic range: ± 8*g*, sampling frequency range: 10–100 Hz [Activinsights, Huntingdon, Cambridgeshire, United Kingdom]); where *g* is equal to the Earth’s gravitational pull. The ActiGraph GT3X+ was initialised with a sampling frequency of 100 Hz using ActiLife software V6.10.2. The GENEActiv monitor was initialised with a sampling frequency of 100 Hz using the GENEActiv PC software V2.2. Both devices were initialised using the same computer.

### Procedures

Following arrival at the laboratory, study procedures were explained to the participants and written informed consent was obtained. The ActiGraph GT3X+ and GENEActiv accelerometers were then attached to both the hip (right side) and wrist (non-dominant). Consequently, a total of 4 devices were worn by each participant (1 ActiGraph GT3X+ on the hip and wrist; and 1 GENEActiv on the hip and wrist) whilst they performed 16 different activities (4 lying positions, 7 sitting postures and 5 upright activities) in a sequential order for 5 minutes each under laboratory-conditions. The start and end time of each activity was observed (using a clock on a computer) and recorded onto a log sheet. A 30 second break was allocated between each activity. Table 1 summarises the sedentary behaviours and light-intensity physical activities undertaken by the participants.

**Table 1:** Summary of sedentary behaviours and light-intensity physical activities.

Posture	Activity	
Lying †	1	Lying flat on back with legs straight
	2	Lying on back with both legs bent
	3	Lying on side with both legs straight
	4	Lying on side with both legs bent
Sitting ‡	5	Sitting on chair whilst watching TV with both feet on floor (knees at 90 degrees)
	6	Sitting on chair whilst watching TV with legs crossed (right leg over left leg)
	7	Sitting on a chair whilst watching TV with right foot resting on left thigh
	8	Sitting on chair whilst watching TV with legs stretched out forwards (feet touching floor)
	9	Sitting on chair whilst watching TV with legs bent backwards underneath chair
	10	Sitting on a chair with some upper body movement (typing a set statement on a computer)
	11	Sitting whilst playing games on a mobile phone
Upright	12	Standing still
	13	Washing pots
	14	Dusting (set area)
	15	Sweeping floor (set area)
	16	Self-paced free-living walk around the room

^†^ During all lying activities, participants were asked to keep their hands straight by their sides

^‡^ During seated activities 5–9, participants were asked to keep their hands on their thighs

The velocity range of the self-paced free-living walk was approximately 3km/h to 4km/h with movement in a forward direction.

### Data Reduction and Processing: Mean Amplitude Deviation (MAD)

The raw acceleration data from the two ActiGraph GT3X+ (100 Hz; .gt3x files) and two GENEActiv (100 Hz; .bin files) devices were downloaded using ActiLife V6.10.2 and GENEActiv PC software V2.2, respectively. For the computation of the MAD metric, the four sets of raw acceleration files were converted to time-stamped .csv files, which were then exported into Stata/IC V13.1 (Stata Corporation, College Station, Texas, USA) for processing and analysis. The laboratory log sheets (with the observed start and end times of each activity) were utilised for identifying each activity in the time-stamped .csv files.

MAD is defined as:

$$Mean\;Amplitude\;Deviation\;\left(MAD\right)≔\frac{1}{n}\times \sum_{i=1}^{n}\left|r_{i}-\bar{r}\right|$$

*where*;

$$r_{i}=\sqrt{x_{i}^{2}+y_{i}^{2}+z_{i}^{2}}=i^{th}\;vector\;magnitude\;at\;each\;time\;point$$

$$\bar{r}=mean\;vector\;magnitude\;within\;the\;time\;period\;of\;interest$$

*n* = *length of the time period*

Each axis (*x*, *y*, *z*) of the raw tri-axial data were first multiplied by 1000 to transform the signals from gravitational units into milligravitational (m*g*) units. This was implemented in order to ensure that the developed thresholds would be comparable with the prior findings in the literature [33]. Research suggests that a 5 second time period can be considered to be adequate for reporting different activities [33, 36]. Therefore, since the accelerometers were initialised at their maximum possible frequencies (100 Hz i.e. 100 samples per second), the length of the time period (*n*) was derived to be 500 (100 Hz x 5 seconds). The vector magnitude (*r*) was calculated at each time point (*i*), followed by the mean vector magnitude for the 5 second time period ($\bar{r}$). This allowed the computation of the MAD metric—which provided a measure of the intensity for every 5 seconds of data.

### Data Reduction and Processing: Euclidean Norm Minus One (ENMO)

For the computation of the ENMO metric, the ActiGraph GT3X+ .gt3x files were converted to time-stamp free .csv files (to avoid computer memory issues, using time-stamp free .csv files is recommended here). The ActiGraph GT3X+ .csv files and the GENEActiv .bin files were then exported into R statistical software V3.1.2 (R Foundation for Statistical Computing, Vienna, Austria, https://cran.r-project.org/) for processing using the GGIR package V1.2–0 which auto-calibrated the raw triaxial accelerometer signals and computed the ENMO metric [30]. The package regenerated the time-stamps and the files were exported into Stata/IC V13.1 for further processing and analysis.

ENMO, described in detail elsewhere [25, 29, 30], is defined as:

$$Euclidean\;Norm\;Minus\;One\;\left(ENMO\right)≔r_{i}-1000$$

*where*;

$$r_{i}=\sqrt{x_{i}^{2}+y_{i}^{2}+z_{i}^{2}}=i^{th}\;vector\;magnitude\;at\;each\;time\;point$$

1000 = 1000 *milligravitational units* = 1 *gravitational unit*

The ENMO subtracts a fixed offset value of 1 gravitational unit at each time point to correct for gravity [25, 29, 30]. Negative ENMO values are rounded up to zero to reduce any bias and error [29, 30]. By design, the ENMO metric is sensitive to poor calibration [30]. Therefore, in order to address these calibration issues, ENMO was calculated using the GGIR package V1.2–0 in R statistical software V3.1.2, which auto-calibrates the raw triaxial accelerometer signal. Further information on the accelerometer calibration technique can be found elsewhere [30]. As with MAD, ENMO was expressed in m*g* and calculated over 5 second epochs.

To ensure the quality of the findings, the first and last 30 seconds of data of each activity were excluded as it was anticipated these time periods might include transitional movements. As a result, only the central 4 minutes of data of each activity were utilised for analysis.

### Statistical Analysis

All statistical analyses were conducted using Stata/IC V13.1. Activities 1 to 11 (any form of lying plus any form of sitting) were combined into one group and classified as ‘sedentary behaviours’. For each activity, the means and standard errors of the ENMO and MAD values stratified by accelerometer brand (ActiGraph GT3X+ and GENEActiv) and monitor placement (hip and wrist) were calculated and tabulated. Unpaired t-tests were implemented in order to compare the mean ENMO and MAD values of each activity between accelerometer brands by monitor placement. Statistical significance was established at p-value<0.05.

Sedentary behaviours were separated from a continuum of light-intensity physical activities ordered by increasing complexity and movement. To achieve this, the following activity discriminations were considered: discrimination 1 = sedentary behaviours vs. standing still, discrimination 2 = sedentary behaviours vs. washing pots, discrimination 3 = sedentary behaviours vs. dusting, discrimination 4 = sedentary behaviours vs. sweeping floor, and discrimination 5 = sedentary behaviours vs. self-paced free-living walk. Univariate binary logistic regression models were fitted with the discrimination under review as the dependent variable and the hip/wrist ENMO/MAD metric as the independent variable. Receiver-Operating-Characteristic (ROC) analyses were implemented to derive the optimum monitor-specific hip and wrist thresholds for activity classification. Performances were summarised by calculating the area under the ROC curves (AUROC) and the thresholds were examined for validity by conducting a leave-one-out-cross-validation (LOOCV). The LOOCV is a model validation technique which assesses the generalizability and performance of a developed model on unseen data [37]. A training (n—1 observations) and testing (1 observation) analysis is implemented (n times, with a different observation left out each time) to estimate the predictive performance of a model. The method works as follows: a model is trained on seen data (n—1 observations) (i.e. the training set) and tested on unseen data i.e. the single observation that was left out (i.e. the testing set). With a different observation left out each time, the model is repeatedly fitted (n times) in order to predict the performance of the model. The performances between the ENMO and MAD metrics were compared using the AUROC and LOOCV AUROC statistics.

All data underlying the findings of this study are included in the following file: S1 File.

## Results

**Fig 1:**
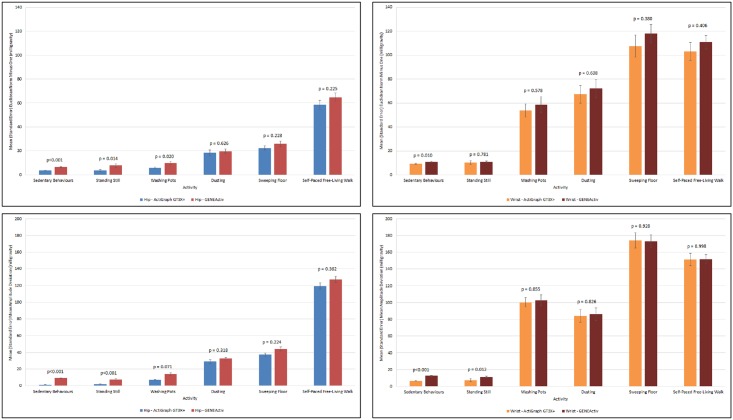
Euclidean Norm Minus One (ENMO) and Mean Amplitude Deviation (MAD) calculated from the ActiGraph GT3X+ and GENEActiv raw acceleration data (MAD) (Top Left: ENMO—Hip, Top Right: ENMO—Wrist, Bottom Left: MAD—Hip, Bottom Right: MAD—Wrist).

The sample consisted of 33 participants [mean age (standard deviation) = 27.4 (5.9) years; mean BMI (standard deviation) = 23.9 (3.7) kg/m^2^; 20 females (60.6%)]. Fig 1, Table 2 (ENMO) and Table 3 (MAD) show the mean (standard error) raw acceleration metric values of the sedentary behaviours and each light-intensity physical activity stratified by accelerometer brand and monitor placement (see S2 File for the mean (standard error) raw acceleration metric values of all the activities). In general, for both hip and wrist monitor placements, the ENMO and MAD values computed from the GENEActiv devices were observed to be slightly higher in comparison to the ActiGraph GT3X+ devices, particularly for the lower-intensity activities. For the monitors positioned on the hip, statistically significant differences (p<0.05) in the ENMO metric were detected between the accelerometer brands during the sedentary behaviours (p<0.001), standing still (p = 0.014) and washing pots (p = 0.020) activities. For the MAD metric, differences were detected during the sedentary behaviours (p<0.001) and standing still (p<0.001) activities. In comparison, for the monitors positioned on the wrist, differences in the ENMO metric were only detected during the sedentary behaviours (p = 0.010). For the MAD metric, differences were detected during the sedentary behaviours (p<0.001) and standing still (p = 0.012) activities.

**Table 2:** Mean (Standard Error) Euclidean Norm Minus One (ENMO (measured in milligravity units)) values for each activity stratified by accelerometer brand and monitor placement.

	ActiGraph GT3X+ (Hip)	GENEActiv (Hip)	
Activity	Mean (Standard Error) ENMO †	Mean (Standard Error) ENMO †	p-value ‡
Sedentary Behaviours	3.6 (0.3)	6.6 (0.3)	<0.001
Standing Still	3.6 (0.7)	7.7 (1.4)	0.014
Washing Pots	5.9 (0.7)	9.8 (1.5)	0.020
Dusting	18.4 (2.2)	19.8 (1.7)	0.626
Sweeping Floor	22.4 (1.8)	25.8 (2.2)	0.228
Self-Paced Free-Living Walk	58.5 (3.7)	64.8 (3.6)	0.225
	ActiGraph GT3X+ (Wrist)	GENEActiv (Wrist)	
Activity	Mean (Standard Error) ENMO †	Mean (Standard Error) ENMO †	p-value ‡
Sedentary Behaviours	9.3 (0.5)	10.9 (0.4)	0.010
Standing Still	10.3 (1.6)	10.8 (0.9)	0.781
Washing Pots	53.8 (5.4)	58.5 (6.6)	0.578
Dusting	67.3 (7.5)	72.3 (7.4)	0.638
Sweeping Floor	107.5 (9.2)	118.0 (7.7)	0.380
Self-Paced Free-Living Walk	103.1 (7.4)	110.9 (5.6)	0.406

Sedentary behaviours = any form of lying plus any form of sitting (activities 1 to 11)

^†^ Euclidean Norm Minus One (ENMO) measured in milligravity units

^‡^ Unpaired t-tests

**Table 3:** Mean (Standard Error) Mean Amplitude Deviation (MAD (measured in milligravity units)) values for each activity stratified by accelerometer brand and monitor placement.

	ActiGraph GT3X+ (Hip)	GENEActiv (Hip)	
Activity	Mean (Standard Error) MAD †	Mean (Standard Error) MAD †	p-value ‡
Sedentary Behaviours	1.2 (0.1)	9.1 (0.8)	<0.001
Standing Still	1.8 (0.3)	7.3 (0.8)	<0.001
Washing Pots	6.9 (0.8)	13.9 (3.7)	0.071
Dusting	29.3 (2.3)	32.8 (2.7)	0.318
Sweeping Floor	37.4 (3.1)	44.2 (4.5)	0.224
Self-Paced Free-Living Walk	119.3 (6.3)	127.3 (6.1)	0.362
	ActiGraph GT3X+ (Wrist)	GENEActiv (Wrist)	
Activity	Mean (Standard Error) MAD †	Mean (Standard Error) MAD †	p-value ‡
Sedentary Behaviours	6.7 (0.5)	12.7 (0.4)	<0.001
Standing Still	7.5 (1.1)	11.3 (1.0)	0.012
Washing Pots	100.2 (8.3)	102.6 (9.8)	0.855
Dusting	84.2 (6.6)	86.2 (6.5)	0.826
Sweeping Floor	174.3 (10.4)	173.0 (10.1)	0.928
Self-Paced Free-Living Walk	151.5 (6.2)	151.6 (6.3)	0.998

Sedentary behaviours = any form of lying plus any form of sitting (activities 1 to 11)

^†^ Mean Amplitude Deviation (MAD) measured in milligravity units

^‡^ Unpaired t-tests

Tables 4 (ENMO; hip), 5 (ENMO; wrist), 6 (MAD; hip) and 7 (MAD; wrist) show the monitor-specific hip and wrist raw acceleration metric thresholds (with the corresponding statistics: metric threshold, sensitivity, specificity, AUROC, LOOCV AUROC) for differentiating between the sedentary behaviours and each light-intensity physical activity. For both hip and wrist monitor placements, poor classification was observed when attempting to isolate standing still from sedentary behaviours (ActiGraph GT3X+ ENMO AUROC [hip: 0.543, wrist: 0.601]; GENEActiv ENMO AUROC [hip: 0.504, wrist: 0.468]; ActiGraph GT3X+ MAD AUROC [hip: 0.638, wrist: 0.603]; GENEActiv MAD AUROC [hip: 0.297, wrist: 0.560]). However, in contrast, sedentary behaviours differentiated well from all motion-based light-intensity physical activities (washing pots, dusting, sweeping floor and self-paced free-living walk; in general, AUROCs >0.95). The LOOCV procedure indicated robustness and stability as the high performance, where observed, was maintained. ENMO and MAD registered similar performances for classifying all motion-based activities for both devices positioned on the hip. However, some small differences between the metrics were observed when distinguishing between sedentary behaviours and standing still. For the accelerometers positioned on the wrist, ENMO and MAD registered comparable performances for both devices (see Tables 4–7).

**Table 4:** Monitor-specific hip Euclidean Norm Minus One (ENMO (measured in milligravity units)) thresholds to differentiate between sedentary behaviours and light-intensity physical activities.

Monitor Placement: Hip		Discrimination				
		Sedentary Behaviours				
		vs.				
		Standing Still	Washing Pots	Dusting	Sweeping Floor	Self-Paced Free-Living Walk
ENMO Threshold (milligravity)	AG	2.6	2.9	7.5	9.4	26.6
	GA	3.9	4.7	8.6	11.2	25.9
Sensitivity (%)	AG	52%	88%	97%	100%	94%
	GA	70%	85%	97%	100%	100%
Specificity (%)	AG	54%	59%	92%	94%	100%
	GA	35%	45%	80%	87%	97%
ENMO AUROC † (95% CI)	AG	0.543 (0.447, 0.640)	0.779 (0.716, 0.842)	0.965 (0.947, 0.982)	0.979 (0.967, 0.992)	0.994 (0.987, 1.000)
	GA	0.504 (0.402, 0.607)	0.666 (0.578, 0.753)	0.934 (0.906, 0.962)	0.961 (0.943, 0.980)	0.997 (0.993, 1.000)
ENMO LOOCV AUROC ‡ (95% CI)	AG	0.382 (0.252, 0.491)	0.689 (0.605, 0.773)	0.962 (0.943, 0.981)	0.978 (0.965, 0.991)	0.993 (0.986, 1.000)
	GA	0.332 (0.212, 0.451)	0.600 (0.499, 0.701)	0.930 (0.900, 0.959)	0.958 (0.939, 0.978)	0.996 (0.992, 1.000)

Sedentary behaviours = any form of lying plus any form of sitting (activities 1 to 11); ENMO = Euclidean Norm Minus One (measured in milligravity units); AG = ActiGraph GT3X+ accelerometer; GA = GENEActiv accelerometer

^†^ Area under Receiver-Operating-Characteristic curve

^‡^ Leave-One-Out-Cross-Validation Area under Receiver-Operating-Characteristic curve

**Table 5:** Monitor-specific wrist Euclidean Norm Minus One (ENMO (measured in milligravity units)) thresholds to differentiate between sedentary behaviours and light-intensity physical activities.

Monitor Placement: Wrist		Discrimination				
		Sedentary Behaviours				
		vs.				
		Standing Still	Washing Pots	Dusting	Sweeping Floor	Self-Paced Free-Living Walk
ENMO Threshold (milligravity)	AG	5.7	25.8	27.9	52.5	41.4
	GA	8.7	30.7	34.4	52.6	47.1
Sensitivity (%)	AG	79%	94%	94%	91%	91%
	GA	70%	97%	100%	100%	100%
Specificity (%)	AG	45%	93%	94%	99%	99%
	GA	43%	99%	99%	100%	100%
ENMO AUROC † (95% CI)	AG	0.601 (0.520, 0.682)	0.965 (0.928, 1.000)	0.963 (0.913, 1.000)	0.952 (0.887, 1.000)	0.966 (0.920, 1.000)
	GA	0.468 (0.381, 0.555)	0.994 (0.986, 1.000)	0.999 (0.996, 1.000)	1.000 (1.000, 1.000)	1.000 (1.000, 1.000)
ENMO LOOCV AUROC ‡ (95% CI)	AG	0.337 (0.238, 0.436)	0.957 (0.907, 1.000)	0.955 (0.896, 1.000)	0.938 (0.855, 1.000)	0.950 (0.886, 1.000)
	GA	0.383 (0.306, 0.451)	0.992 (0.981, 1.000)	0.998 (0.994, 1.000)	1.000 (1.000, 1.000)	1.000 (1.000, 1.000)

Sedentary behaviours = any form of lying plus any form of sitting (activities 1 to 11); ENMO = Euclidean Norm Minus One (measured in milligravity units); AG = ActiGraph GT3X+ accelerometer; GA = GENEActiv accelerometer

^†^ Area under Receiver-Operating-Characteristic curve

^‡^ Leave-One-Out-Cross-Validation Area under Receiver-Operating-Characteristic curve

**Table 6:** Monitor-specific hip Mean Amplitude Deviation (MAD (measured in milligravity units)) thresholds to differentiate between sedentary behaviours and light-intensity physical activities.

Monitor Placement: Hip		Discrimination				
		Sedentary Behaviours				
		vs.				
		Standing Still	Washing Pots	Dusting	Sweeping Floor	Self-Paced Free-Living Walk
MAD Threshold (milligravity)	AG	0.8	2.8	7.4	17.8	33.2
	GA	7.2	8.5	16.2	18.4	35.0
Sensitivity (%)	AG	70%	94%	100%	100%	100%
	GA	33%	67%	97%	100%	100%
Specificity (%)	AG	52%	89%	100%	100%	100%
	GA	38%	69%	99%	100%	100%
MAD AUROC † (95% CI)	AG	0.638 (0.545, 0.732)	0.958 (0.927, 0.989)	1.000 (1.000, 1.000)	1.000 (1.000, 1.000)	1.000 (1.000, 1.000)
	GA	0.297 (0.206, 0.387)	0.710 (0.602, 0.818)	0.986 (0.973, 0.999)	1.000 (1.000, 1.000)	1.000 (1.000, 1.000)
MAD LOOCV AUROC ‡ (95% CI)	AG	0.584 (0.479, 0.690)	0.954 (0.917, 0.990)	1.000 (1.000, 1.000)	1.000 (1.000, 1.000)	1.000 (1.000, 1.000)
	GA	0.278 (0.191, 0.365)	0.653 (0.517, 0.789)	0.975 (0.928, 0.998)	1.000 (1.000, 1.000)	1.000 (1.000, 1.000)

Sedentary behaviours = any form of lying plus any form of sitting (activities 1 to 11); MAD = Mean Amplitude Deviation (measured in milligravity units); AG = ActiGraph GT3X+ accelerometer; GA = GENEActiv accelerometer

^†^ Area under Receiver-Operating-Characteristic curve

^‡^ Leave-One-Out-Cross-Validation Area under Receiver-Operating-Characteristic curve

**Table 7:** Monitor-specific wrist Mean Amplitude Deviation (MAD (measured in milligravity units)) thresholds to differentiate between sedentary behaviours and light-intensity physical activities.

Monitor Placement: Wrist		Discrimination				
		Sedentary Behaviours				
		vs.				
		Standing Still	Washing Pots	Dusting	Sweeping Floor	Self-Paced Free-Living Walk
MAD Threshold (milligravity)	AG	4.2	33.4	35.9	73.4	66.1
	GA	10.6	39.6	45.2	74.5	67.1
Sensitivity (%)	AG	73%	100%	100%	100%	100%
	GA	45%	100%	100%	100%	100%
Specificity (%)	AG	55%	98%	98%	100%	100%
	GA	58%	98%	99%	100%	100%
MAD AUROC † (95% CI)	AG	0.603 (0.512, 0.694)	0.999 (0.998, 1.000)	0.998 (0.996, 1.000)	1.000 (1.000, 1.000)	1.000 (1.000, 1.000)
	GA	0.560 (0.447, 0.673)	0.999 (0.998, 1.000)	0.998 (0.996, 1.000)	1.000 (1.000, 1.000)	1.000 (1.000, 1.000)
MAD LOOCV AUROC ‡ (95% CI)	AG	0.372 (0.264, 0.479)	0.996 (0.990, 1.000)	0.998 (0.995, 1.000)	1.000 (1.000, 1.000)	1.000 (1.000, 1.000)
	GA	0.441 (0.346, 0.535)	0.998 (0.996, 1.000)	0.998 (0.995, 1.000)	1.000 (1.000, 1.000)	1.000 (1.000, 1.000)

Sedentary behaviours = any form of lying plus any form of sitting (activities 1 to 11); MAD = Mean Amplitude Deviation (measured in milligravity units); AG = ActiGraph GT3X+ accelerometer; GA = GENEActiv accelerometer

^†^ Area under Receiver-Operating-Characteristic curve

^‡^ Leave-One-Out-Cross-Validation Area under Receiver-Operating-Characteristic curve

## Discussion

This is the first methodological study to develop and validate ENMO and MAD intensity-based thresholds for differentiating between sedentary behaviours and common light-intensity physical activities using raw acceleration data collected from both hip- and wrist-worn ActiGraph GT3X+ and GENEActiv tri-axial accelerometers.

The monitor-specific hip and wrist ENMO and MAD thresholds showed excellent ability for differentiating between the sedentary behaviours and motion-based light-intensity physical activities (washing pots, dusting, sweeping floor and self-paced free-living walk). Poor classification was experienced when attempting to isolate standing still from sedentary behaviours. However, these findings are as expected since the magnitude of the acceleration signals is very similar when lying/sitting or standing still, and in order to accurately discriminate between postures, more features of the acceleration signal (such as the angles between the individual orthogonal axes of acceleration from wrist-worn accelerometers) need to be considered [11, 38, 39]. Recent experimental and epidemiological research has shown that standing can have beneficial effects on health [12–14]. However, due to their poor performances, the thresholds developed in this study to discriminate between sedentary behaviours and standing would miscalculate the times spent in each characteristic (i.e. they would overestimate sedentary time and underestimate standing time). Whilst taking into consideration that this issue categorically depends on the amount of time individuals actually spend standing still in day-to-day life, adequate caution should be taken if isolating sedentary behaviours from standing is of particular interest using these thresholds. In contrast, the magnitude of the acceleration signals is considerably larger during standing activities which require some light movement (e.g. washing pots) and light-intensity lateral/anteroposterior activities (e.g. dusting; sweeping floor, etc.), and as shown, they can be separated well from sedentary behaviours using both the ENMO and MAD metrics. In comparison to the hip ENMO/MAD values and thresholds, the wrist ENMO/MAD values and thresholds were higher during the light-intensity physical activities, reflecting the supplementary arm/hand movements whilst performing these particular activities. Findings were robust as the observed performances were widely sustained. The ENMO and MAD metrics tended to perform similarly across activities and accelerometer brands.

Hildebrand and colleagues [29] recently developed monitor-specific hip and wrist ENMO thresholds for moderate and vigorous physical activities using raw acceleration data from the ActiGraph GT3X+ and GENEActiv devices. ENMO was fairly comparable between the two accelerometer brands in adults, but not in children. The MAD thresholds developed by Vähä-Ypyä and colleagues were much larger in comparison to our monitor-specific thresholds as they predominantly focused on classifying higher intensity activities. [33]. However, to our knowledge, until our study, no thresholds to separate sedentary behaviours from light-intensity physical activities existed for any raw acceleration metric for these two devices under either wear-site. Via using the abundance of high performing thresholds (based on different metrics, accelerometer brands and wear-sites) generated in this study, researchers can now accurately separate sedentary behaviours from common motion-based activities—with all the thresholds developed for washing pots recommended to be used as potential proxy indicators of entering and engaging in light-intensity physical activity, irrespective of the metric, accelerometer brand and wear-site used.

For both hip and wrist monitor placements, in comparison to the ActiGraph GT3X+ monitors, the ENMO and MAD values derived from the GENEActiv devices were observed to be slightly higher, particularly for the lower-intensity activities. A greater magnitude of accelerations from the GENEActiv has been previously reported when conducting time domain analyses of raw acceleration data using these two accelerometers [22, 23]. Extensive mechanical testing (e.g. shaker diagnostics) has revealed that the differences observed in the magnitude of the raw acceleration signals (e.g. vector magnitude) between the two devices (GENEActiv established to have higher accelerations) could be due to underlying inner structural variances [22, 23], with features such as the MEMS (microelectromechanical systems) sensor affecting the signal processing and digitization of the output. Other potential causal factors include; the zero-g offset, reference voltage, analog-to-digital bit-rate conversion, any proprietary filtering, and sensitivity of the MEMS sensor [22, 40]. Methods for minimizing the differences between the devices include the applications of affine conversions (e.g. correction factors) to the raw acceleration signals [23]. It appears that features from the frequency domain are comparable between the two accelerometers [23], although these methods are more intricate. In theory, raw acceleration data between different devices should be comparable to each other. However, this study further highlights the subtle differences that become apparent when comparing monitors.

Essentially, both the ENMO and MAD metrics offer simple ways of correcting for the gravitational component. Bassett and colleagues recommend that any newly proposed raw acceleration system should be compared to the ones already in use [41]. Although Vähä-Ypyä and colleagues compared the performance of MAD to several other raw acceleration traits (where it proved to be the most exemplary method) [33], it was not equated to the ENMO metric. ENMO is emerging as the prototypical trait for analysing raw acceleration data and is being widely used by accelerometer researchers [25, 29, 30], thus, following recommendations from Bassett and colleagues [41], it is imperative to compare MAD to ENMO. Furthermore, since the ENMO metric is sensitive to poor calibration of the accelerometer [30], it is ideal to implement sensor calibrated ENMO in order to reduce any erroneous findings. Our study indicates that in addition to ENMO, MAD provides an alternative, yet a robust and straightforward technique for analysing raw acceleration data.

### Strengths and Limitations

Our study has several strengths and some limitations. Strengths include; utilisation of widely-used accelerometers; comprehensive data analysis of raw acceleration data collected at a high sampling frequency; laboratory-based experimental design with usage of both hip- and wrist-worn devices; robust statistical analysis; and the generation of monitor-specific thresholds that distinguish between sedentary and non-sedentary activities. These thresholds are sample and protocol specific, implying that the classifications of sedentary behaviours and light-intensity physical activities should be broadly comparable between studies—irrespective of the metric, accelerometer brand and wear-site used. However, these strengths also have some inherent limitations. The laboratory-based settings may limit generalizability to free-living environments. In particular, it may be more difficult to classify sedentary behaviours in free-living situations using wrist-worn devices since individuals can carry out sedentary tasks involving arm movement as highlighted in this study (e.g. using a computer and a mobile phone whilst in a seated position). Secondly, with the participants instructed to keep their hands on their thighs during the seated activities that involved watching TV (sitting activities 5–9), the real-life positioning and postures of these sedentary behaviours are less likely to be reflected or fully captured. Nevertheless, for both the accelerometer brands, determining the wrist ENMO and MAD thresholds using computer activity as the only sedentary behaviour did not affect the performance or value of the thresholds (in general, AUROCs >0.95 with validation indicating robustness and <10% change in threshold values (data not shown)). Furthermore, the limited range of sedentary behaviours and light-intensity physical activities can be considered as a weakness of the study. The inclusion of additional activities, such as eating, reading a book or using a mobile phone whilst standing, would have added to the strengths. Lastly, although the thresholds in this study were validated, they were only done so internally. Therefore, it is desirable to cross-validate all available ENMO and MAD thresholds externally; the performance of algorithms developed in laboratory conditions attenuates when applied in field environments [42].

### Conclusions

In conclusion, the ENMO and MAD metrics are accessible and increasingly used approaches for analysing hip- and wrist-worn raw accelerometer data, however, well-developed and validated methods utilising outputs from these methods are sparse. Our study provides comprehensible monitor-specific hip and wrist ENMO and MAD thresholds for analysing raw acceleration data, particularly for researchers interested in sedentary behaviour and light-intensity movement. Users can exploit these robust ENMO and MAD thresholds in order to accurately separate sedentary behaviours from common motion-based light-intensity physical activities. However, caution should be taken if separating sedentary behaviours from standing is of specific interest.

In terms of making recommendations for future research, due to its’ proven competency and continuous use in the field, this study further supports the use of sensor calibrated ENMO, a well-performing metric which is emerging as the prototypical tool for the analysis of raw acceleration data, to help promote comparability between studies. Nevertheless, the MAD metric also offers an alternative, robust and straightforward technique for analysing raw acceleration data; however, more studies further exploring the MAD metric are required.

## Supporting Information

S1 File Supporting Information—S1, Bakrania and colleagues. (XLSX)

S2 File Supporting Information—S2, Bakrania and colleagues. (XLSX)
